# Why Hepatic CYP2E1-Elevation by Itself Is Insufficient for Inciting NAFLD/NASH: Inferences from Two Genetic Knockout Mouse Models

**DOI:** 10.3390/biology9120419

**Published:** 2020-11-26

**Authors:** Maria Almira Correia, Doyoung Kwon

**Affiliations:** 1 Departments of Cellular & Molecular Pharmacology, Pharmaceutical Chemistry, and Bioengineering and Therapeutic Sciences, and The Liver Center, University of California San Francisco, San Francisco, CA 94158-2517, USA; 2 Departments of Cellular & Molecular Pharmacology, University of California San Francisco, San Francisco, CA 94158-2517, USA; doyoung.kwon@pusan.ac.kr

**Keywords:** CYP2E1, E3 ubiquitin ligase, gp78/AMFR, CHIP, ubiquitin-dependent proteasomal degradation (UPD), NAFLD/NASH

## Abstract

**Simple Summary:**

Cytochrome P450 CYP2E1 is an enzyme engaged in the breakdown of various compounds (drugs, carcinogens, dietary nutrients and toxins) as well as endogenous compounds (steroids and fatty acids), resulting in both detoxification and elimination of these agents. Often, it can also convert these agents, i.e., Acetaminophen, into more toxic products. Elevated liver CYP2E1 content is implicated in various metabolic diseases including alcoholic liver disease, nonalcoholic fatty liver disease (NAFLD)/nonalcoholic steatohepatitis (NASH), diabetes and obesity. While liver CYP2E1 elevation is considered essential to the pathogenesis of these liver diseases, our findings of elevated liver CYP2E1 content in two genetic mouse models with impaired degradation and thus a disrupted disposal of CYP2E1 when fed a regular lab chow diet, argue that it is not sufficient for triggering NAFLD/NASH. Thus, despite comparable hepatic CYP2E1 elevation and functional stabilization in these two models, NAFLD/NASH was only observed in the mouse livers that exhibited concurrently enhanced liver fat production. These findings reinforce the notion that in addition to elevated liver CYP2E1 content, CYP2E1-mediated NAFLD/NASH requires liver fat accumulation derived from either enhanced liver fat-production or ingestion of a high fat/high carbohydrate diet.

**Abstract:**

Hepatic cytochrome P450 CYP2E1 is an enzyme engaged in the metabolic biotransformation of various xenobiotics and endobiotics, resulting in both detoxification and/or metabolic activation of its substrates to more therapeutic or toxic products. Elevated hepatic CYP2E1 content is implicated in various metabolic diseases including alcoholic liver disease, nonalcoholic fatty liver disease (NAFLD)/nonalcoholic steatohepatitis (NASH), diabetes and obesity. While hepatic CYP2E1 elevation is considered essential to the pathogenesis of these liver diseases, our findings in two mouse models of E3 ubiquitin ligase genetic ablation fed a regular lab chow diet, argue that it is not sufficient for triggering NAFLD/NASH. Thus, albeit comparable hepatic CYP2E1 elevation and functional stabilization in these two models upon E3 ubiquitin ligase genetic ablation and consequent disruption of its ubiquitin-dependent proteasomal degradation, NAFLD/NASH was only observed in the mouse livers that exhibited concurrent SREBP1c-transcriptional upregulation of hepatic lipogenesis. These findings reinforce the critical complicity of an associated prolipogenic scenario induced by either an inherently upregulated hepatic lipogenesis or a high fat/high carbohydrate diet in CYP2E1-mediated NAFLD/NASH.

## 1. Introduction

Human liver CYP2E1 is an endoplasmic reticulum (ER)-anchored cytochrome P450 involved in the biotransformation of clinically relevant drugs (acetaminophen, halothane), ethanol and other xenobiotics, carcinogens (nitrosamines), endogenous acetone and fatty acids (arachidonic acid) to toxic/reactive intermediates [1,2,3,4]. Despite its relatively low hepatic abundance (≈5–7% of human hepatic P450 content), its ability to bioactivate xenobiotics into toxic/reactive intermediates and its high propensity for inciting oxidative stress have implicated CYP2E1 in the pathogenesis of toxic liver damage, alcoholic liver disease, nonalcoholic steatohepatitis (NASH), diabetes and obesity [5,6,7,8,9,10,11,12,13,14]. Its abnormally elevated basal content (>7%) either via transcriptional induction or protein stabilization in these conditions is thought to predispose and/or abet pathogenesis of liver injury [1,5,6,7,8,9,10,11,12,13,14]. Specifically, CYP2E1 overexpression has long been causally associated with both alcoholic fatty liver disease, as well as non-alcoholic fatty liver disease (NAFLD) [2,3,4,5,6,7,8,9,10,11,12,13,14]. In obese and/or Type II diabetic humans as well as dietary NAFLD rodent models, such CYP2E1 overexpression has been invariably linked to insulin resistance and the progression of NAFLD to NASH (non-alcoholic steatohepatitis) [10,12,13,14]. A key feature of this CYP2E1-mediated progression is the enzyme’s intrinsic catalytic propensity to undergo futile catalytic cycles in the absence of substrates, engendering reactive oxygen species (ROS) that can attack vicinal accumulated fats and trigger lipid peroxidation and oxidative stress [15,16,17]. In turn, ROS/oxidative stress activate the hepatic c-Jun-N-terminal kinase (JNK)-signaling via the apoptosis signaling kinase 1 (ASK1)-mitogen activated protein kinase kinase (MAPKK) signaling cascade, with consequent JNK-mediated Ser307-phosphorylation of insulin receptor substrates, IRS-1 and IRS-2 [18,19,20]. Such JNK-mediated Ser307-phosphorylation of IRS-1 and IRS-2 apparently impairs their Tyr895-phosphorylation that normally results in insulin interaction with its hepatic cell-surface receptor, thus reducing insulin signaling-elicited Akt-activation via Thr308/Ser473 phosphorylation [19,20,21]. Reduced Akt-activation in turn results on the one hand, in the impaired activation of glycogen synthase kinase 3 (GSK3)-Kinase which inactivates GSK3, an inactivation required to switch on glycogenesis [20,21,22,23]; and on the other, in the impaired phosphorylation and consequently reduced nuclear export of hepatic FOXO1/3 factors required to limit their transcriptional activation of the nuclear gluconeogenic enzyme, PEPCK [24,25,26]. Thus, CYP2E1-elicited ROS and oxidative stress by impairing insulin-signaling would result in increased gluconeogenesis.

Similar observations have also been documented in CYP2E1-overexpressing hepatocyte cell lines (RALA255-10G, HepG2) apparently cultured in regular cell culture media, not particularly enriched in fatty acids or other lipogenic sources [8,12,27]. In HepG2 cells, CYP2E1-stabilization upon 3-methyladenine-mediated inhibition of their autophagic pathway, also potentiated arachidonic acid-elicited oxidative stress and cytotoxicity [28].

Intriguingly, we encountered a similar pathogenic scenario in mice upon genetic ablation of the E3 ubiquitin (Ub)-ligase C-terminus of Hsc70-interacting protein (CHIP) [29], an essential participant in the Ub-dependent proteasomal degradation (UPD) of hepatic CYP2E1 [30,31]. This CHIP-knockout (CHIP^−/−^) resulted in a hepatic ≈180–200% stabilization of CYP2E1 ER-content in these mice [29]. This CYP2E1 stabilization in CHIP^−/−^-mice was associated not only with a correspondingly increased functional activity, but also with increased lipid peroxidation with concomitantly increased 4-hydroxynonenal tissue conjugates and 15-F_2T_-isoprostane levels, marked activation of the hepatic JNK-cascade and microvesicular fat accumulation quite early at 2 months, which within 8–9 months of age progressed to macrovesicular fat accumulation, hepatocyte ballooning and injury, typical of clinical NASH [29]. Surprisingly, this occurred in spite of the fact that these CHIP^−/−^-mice had been fed a regular, non-fat/carbohydrate-enriched chow-diet, suggesting that overexpression of hepatic CYP2E1 content and consequent oxidative stress were sufficient for NASH-development [29]. This finding seemingly contradicted previous findings [32] that hepatic CYP2E1 induction upon administration of the drug isoniazid (INH) to rats had failed to increase their in vivo hepatic F-isoprostane levels, generally recognized as very sensitive and reliable markers of lipid peroxidation, indicating either that CYP2E1 induction alone was insufficient for eliciting lipid peroxidation and consequent oxidative stress, or that INH while inducing CYP2E1 expression had inhibited its potential to trigger lipid peroxidation via competitive substrate inhibition.

Puzzled by these findings, we examined whether a comparable hepatic CYP2E1 stabilization observed upon liver-specific genetic ablation of gp78/AMFR (autocrine motility factor receptor), an ER-polytopic E3 Ub-ligase also known to participate in CYP2E1-UPD [30,33], would mimic the findings in CHIP^−/−^-mice [29]. Indeed, we found a comparable stabilization of hepatic CYP2E1 content and function in gp78^−/−^-mice relative to wild type (WT) mice [33]. However, by contrast to CHIP^−/−^-mouse hepatocytes, gp78^−/−^-mouse hepatocytes, albeit from comparably age-matched mice similarly fed dietary chow, exhibited no comparable elevation of hepatic lipid peroxidation, fatty acids and/or triglyceride content over corresponding age-matched WT-controls [34]. These differential findings in CHIP^−/−^-mice versus gp78^−/−^-mice suggested that factors additional to elevated hepatic CYP2E1 content most likely contributed to the early microvesicular hepatic fat accumulation observed in CHIP^−/−^-mice.

**Figure 1:**
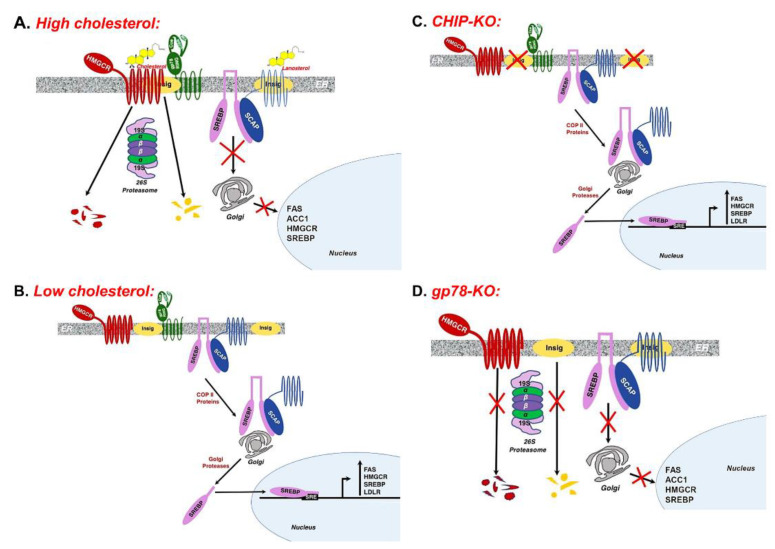
The hepatic INSIG-SCAP-SREBP1 axis and its activation under certain conditions. (**A**) Under high sterol levels, the endoplasmic reticulum (ER)-polytopic HMGCR, the rate-limiting enzyme in sterol synthesis, associates with the ER-integral Insig-gp78 complex, resulting in the gp78-mediated ERAD/UPD of both HMGCR and Insigs. High sterol levels also enhance the association of the SREBP-SCAP-INSIG complex resulting in their ER-retention and preventing the transcriptional activation of lipogenesis and steroidogenesis. (**B**) When sterol levels drop, SREBP-SCAP dissociates from the Insigs are escorted by COPII proteins to the Golgi wherein the precursor SREBPs are N-terminally processed by Golgi proteases into their corresponding transcriptionally active nuclear species. The nuclear SREBP species activates the transcription of various genes including lipogenic (FAS, ACC1), steroidogenic (HMGCR) as well as SREBP genes. (**C**) Upon CHIP-KO, drop of Insig ER-levels possibly due to insulin resistance, release SREBP-SCAP from the ER with their subsequent transport to the Golgi and processing into the transcriptionally active forms, resulting in enhanced hepatic lipogenesis and steroidogenesis. (**D**) Upon gp78-KO, by contrast, HMGCR and Insigs are stabilized in the ER, leading to the retention of the SREBP-SCAP complex within the ER and consequent abortion of the SREBP proteolytic processing into the transcriptionally active nuclear species. Figures were based on literature reports [29,34,35,36,37,38,39,40,41,42,43,44,45,46]. HMGCR: HMG CoA Reductase; Insig: Insulin-induced gene; SCAP: SREBP-Cleavage Activating Protein; SREBP: Sterol Regulatory Element Binding Protein.

In search of clues, we focused on the hepatic Insig 1/2-SREBP1-SCAP ER-complex involved in lipogenic regulation [35,36,37,38]. The glucose- and insulin-regulated sterol regulatory element binding proteins SREBPs (SREBP-1 and SREBP-2) are key lipogenic transcription factors that are normally complexed to SCAP (SREBP-Cleavage Activating Protein) [37,38]. When cellular sterols are plentiful, SREBPs are sequestered in the ER through their association with the ER-integral insulin-induced scaffold proteins Insigs 1 and 2 (Figure 1A). When cellular sterol levels drop (Figure 1B), dissociation of the Insig-SREBP-SCAP ER-complex is required for SCAP to escort the precursor SREBP species in a COPII protein-mediated transport pathway to the Golgi for its sequential N-terminal proteolytic processing by two membrane-bound proteases into the mature transcriptionally active N-terminal basic-helix-loop-helix (bHLH)-Zip SREBP species into the cytosol and thence into the nucleus ([37,38,39,40,41] Figure 1B). Whereas nuclear SREBP-2 species is largely involved in regulating cellular cholesterol homeostasis through activation of genes involved in cholesterol synthesis, metabolism and uptake, nuclear SREBP-1c species is predominantly involved in regulating lipogenesis through transcriptional activation of genes involved in fatty acid synthesis and triglyceride formation, including SREBP-1 itself [38,39,40,41].

In CHIP^−/−^-mice, we found that hepatic Insig-1 and Insig-2 mRNA expression albeit comparable at 2 months of age, markedly decreased at 9 months of age relative to corresponding WT [29]. By contrast, hepatic SREBP-1c mRNA expression showed the opposite response: although slightly increased at 2 months, it was significantly increased at 9 months of age, relative to corresponding age-matched WT [29]. This was as expected paralleled by significantly increased hepatic *fas1* and *scd-1* mRNA expression at 2 months with further increases at 9 months of age [29]. Furthermore, immunoblotting analyses of CHIP^−/−^-mouse hepatocytes revealed not only a decrease in hepatic Insig-1 (36% of WT levels) and Insig-2 (42% of WT levels) proteins, but also a corresponding increase in precursor (208% of WT levels) and nuclear (151% of WT levels) SREBP-1 protein species ([34]; Table 1). This reduction in Insig levels thus accounts for the increased processing of the transcriptionally active SREBP-1 with consequently increased hepatic lipogenesis and progressively increased fat accumulation detected in CHIP^−/−^-mice fed a regular, non-fat/carbohydrate enriched chow diet, starting from 2 months of age on ([29]; Table 1; Figure 1C). Because SREBP-1 autoregulates itself, transcriptional SREBP-1 activation also led to detectable increases in precursor SREBP-1 levels ([29,34]; Table 1).

In contrast, quite the reverse response of the hepatic Insig 1/2-SREBP1-SCAP ER-complex was observed in mice upon liver-specific gp78-ablation ([34]; Table 1 and Figure 1D). Because Insigs are well recognized substrates of gp78-mediated ubiquitination [42,43,44], their cellular degradation (via UPD) is impaired in gp78^−/−^-mice, resulting in a >3-fold ER-enrichment of hepatic Insig-1/2 content ([34]; Table 1 and Figure 1D). This hepatic Insig ER-enrichment in turn led to the sequestration of the SREBP-1-SCAP complex within the ER (Figure 1D), with a resultant small decrease in the precursor (82% of WT-levels) and nuclear (72% of WT-levels) SREBP-1 species ([34]; Table 1). Consequently, the transcriptional activation of hepatic lipogenic genes was, if at all, reduced relative to that of the WT, or even CHIP^−/−^-mouse hepatocytes, and thus, relatively little hepatic lipid peroxidation and microvesicular fat accumulation was observed in gp78^−/−^-mouse hepatocytes, even upon a lipogenic fructose challenge [34]. Furthermore, insulin-signaling as judged by Ser473/Thr308 Akt-phosphorylation and N-terminal FOXO1/3/4-Thr-phosphorylation in these gp78^−/−^-mouse hepatocytes was elevated above that normally observed in corresponding age-matched WT-mouse hepatocytes [34]. Thus, albeit a comparable elevation of hepatic CYP2E1 content and function, gp78^−/−^-mice exhibited an above normal insulin-signaling response and were spared the lipogenic scenario that progressively leads to NAFLD/NASH in CHIP^−/−^-mice [29,34].

## 2. Conclusions

In summary, these findings reveal that the elevation of hepatic CYP2E1 content, while essential to the pathophysiologic process [10,12,13,14], does not by itself trigger NAFLD/NASH. CYP2E1-mediated incitement of NAFLD/NASH requires the absolutely necessary complicity of elevated hepatic lipids, accumulated either through enhanced hepatic lipogenesis or lipogenic diets. Yet, it is noteworthy, that in spite of such a conspiring scenario observed at early age (2 months) in CHIP^−/−^-mice, the hepatic microvesicular steatosis did not rapidly progress to fulminant macrovesicular steatosis typical of NASH, until 8–9 months of age, largely due to the restraint exerted by two concurrent beneficial events: (1) The transcriptional upregulation of hepatic adiponectin receptors R1/R2 and adipose adipoQ gene, along with the concomitant stabilization of AMP-Kinase AMPKα1 (that we identified as a CHIP-target [29]), its ROS-elicited activation, and the consequently enhanced hepatic adiponectin-AMPK-FOXO-signaling that resulted in enhanced hepatic lipophagy and mitochondrial lipid catabolism [29]; and (2) AMPK-mediated phosphorylation of hepatic Insigs-T222 [45] and of SREBP-Ser372 [46], both of which would impair SREBP-transcriptional activation and lipogenesis. It was only when AMPK-mediated signaling succumbed to progressive ROS/JNK-mediated hepatocellular injury in the CHIP^−/−^-mouse liver, that the microvesicular steatosis yielded to the full-blown pathognomonic signs of NASH [29].

## Figures and Tables

**Table 1:** Relative hepatic CYP2E1-, Insig 1/2- and SREBP1c-content and MDA-levels of CHIP- and gp78-knockout (KO) mice.

Parameter	CHIP		gp78	
	WT	KO	WT	KO
CYP2E1	100	213.0	100	155.7 ^b^
MDA	100 ± 3.4 ^a^	169.9 ± 13.9 ^a^	100 ± 2.2	109.9 ± 4.7
Insig-1	100	36.2	100	329.4
Insig-2	100	41.5	100	346.1
SREBP1 (P)	100	208.4	100	82.2
SREBP1 (N)	100	151.4	100	72.9

Hepatocytes were isolated from livers of wild type (WT_ and corresponding CHIP^−/−^ mice or WT and corresponding gp78^−/−^ mice, aged 8–9 weeks, and cultured for 48 h before treatment with the CYP2E1 inducer INH for the next 72 h. Lysates were prepared and aliquots subjected to Western immunoblotting analyses with GAPDH as the loading control. Malondialdehyde (MDA) levels were monitored as described as markers of hepatic lipid peroxidation. Values (Mean of 2 individual mouse livers) were normalized to the GAPDH content and expressed as % of the corresponding WT-content. P, N, precursor and nuclear species, respectively, identified on the basis of their relative molecular masses. Original immunoblotting data were reported [34]. ^a^ MDA-levels in similarly cultured WT and CHIP^−/−^-mouse hepatocytes are included for reference and were derived from our previous report [29]. ^b^ Although the mean CYP2E1-content in the gp78^−/−^-hepatocytes used to concurrently monitor Insig1/2- and SREBP-content was lower than in CHIP^−/−^-hepatocytes, this pertained largely to these two sets of 2 animals each examined concurrently. However, the bulk of our data in our other studies [29,33] revealed the hepatic CYP2E1 content to be quite comparable in both KO-models, ranging between 155.7 and 221.3% of corresponding WT-controls in gp78^−/−^-hepatocytes.
